# Recurrent Myocarditis Following COVID-19 Infection and the mRNA Vaccine

**DOI:** 10.7759/cureus.26650

**Published:** 2022-07-07

**Authors:** Mohammed Afraz Pasha, Sangeetha Isaac, Zubair Khan

**Affiliations:** 1 Department of Internal Medicine, North Alabama Medical Center, Florence, USA; 2 Department of Cardiovascular Disease, North Alabama Medical Center, Florence, USA

**Keywords:** post vaccination myocarditis, cardiac imaging-mri, covid-induced myocarditis, covid-19 vaccination, covid-19

## Abstract

COVID-19 infection has cardiovascular manifestations such as acute myocarditis, arrhythmia, ischemic cardiomyopathy, heart failure, pericardial effusion, cardiac tamponade, and thromboembolism. The COVID-19 mRNA vaccines BNT162b2 (Pfizer-BioNTech), mRNA-1273 (Moderna), and viral vector vaccine Ad26.COV2.S (Johnson & Johnson - Janssen) were initially approved for emergency authorized use by the US-FDA. Cases of myocarditis were reported primarily in adolescents and young adults after administration of COVID-19 mRNA vaccines, with the subsequent emergence of cases of myocarditis after administration of viral vector vaccine Ad26.COV2.S. A majority of these cases were observed after the second dose of the mRNA vaccine. This case report demonstrates the occurrence of symptomatic myocarditis in a patient during acute COVID-19 infection, followed by recurrence of symptoms after the first dose of mRNA COVID-19 vaccine and subsequent recurrence of cardiac MRI-proven myocarditis after the second dose of mRNA COVID-19 vaccine. This case stands out due to the occurrence of symptoms with COVID-19 infection and after vaccination, suggesting possible incomplete interval resolution of infection-related myocarditis.

## Introduction

The COVID-19 pandemic has claimed more than 6 million lives to date [1]. The role of COVID-19 infection in life-threatening cardiovascular sequelae like acute myocarditis, arrhythmia, ischemic cardiomyopathy, heart failure, pericardial effusion, cardiac tamponade, and thromboembolism is well established [2,3]. Immunization against an infectious disease plays a crucial role in prevention and spread. After their development in the laboratory, vaccines undergo serial levels of trials before being approved safe for wide-scale public use. In December 2020, the United States Food and Drug Administration (US-FDA) issued emergency use authorizations for use of BNT162b2 (Pfizer-BioNTech) and mRNA-1273 (Moderna) vaccines for the prevention of COVID-19 infection. Two months later, the Ad26.COV2.S vaccine (Johnson & Johnson - Janssen) became the third vaccine to receive emergency use authorization by the US-FDA. BNT162b2 and mRNA-1273 COVID-19 vaccines are mRNA vaccines requiring two doses, with an additional third booster dose in moderate to severely immunocompromised individuals, while the Ad26.COV2.S COVID-19 vaccine is a single-dose viral vector vaccine. Myocarditis and pericarditis have been reported as potential adverse effects after both mRNA and viral vector vaccines [4]. 

In April 2021, 62 cases of myocarditis following the BNT162b2 COVID-19 vaccine were reported in Israel. Around the same time, 14 cases of myocarditis were also reported in the United States military personnel who received the BNT162b2 COVID-19 vaccine [5]. According to the vaccine adverse event reporting system (VAERS), myocarditis is a rare adverse effect post-vaccination previously reported after the small-pox vaccine. Since April 2021, cases of myocarditis in adolescents and young adults were increasingly reported following the administration of COVID-19 vaccines [6]. However, the causal relationship and mechanism are yet to be established. We report a case of symptomatic myocarditis after COVID-19 infection with subsequent flare-ups after each dose of the mRNA-1273 vaccine.

## Case presentation

A 45-year-old African American male presented to the emergency room with ongoing retrosternal chest pain for two days unrelated to posture or exertion. He did not report any other associated symptoms. He had received his second dose of mRNA-1273 COVID-19 vaccine two days prior to the onset of chest pain. He reported being infected with the SARS-CoV-2 virus, three months prior to his current presentation. He had no significant respiratory symptoms at the time but endorsed similar intermittent chest discomfort lasting for two weeks afterward. He self-quarantined and did not seek medical attention at the time, with spontaneous resolution of symptoms. He subsequently received the first dose of mRNA-1273 COVID-19 vaccine eight weeks later and had a recurrence of similar chest discomfort, 48 hours after the vaccine dose, which was mild and self-resolved over the next two days. The patient had no significant past medical history and was not on any prescription medications. He reported being active and following a daily exercise routine. His only cardiac risk factor was a positive family history of ischemic heart disease in his father at the age of 68.

**Figure 1:**
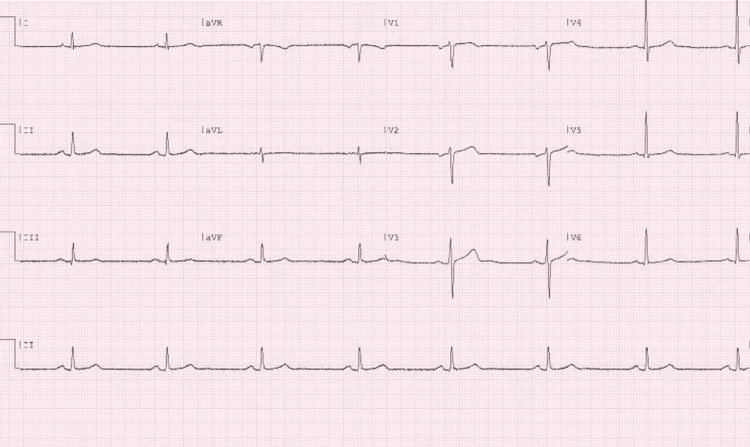
ECG showing sinus bradycardia at 45 bpm without any ST-T changes

**Figure 2:**
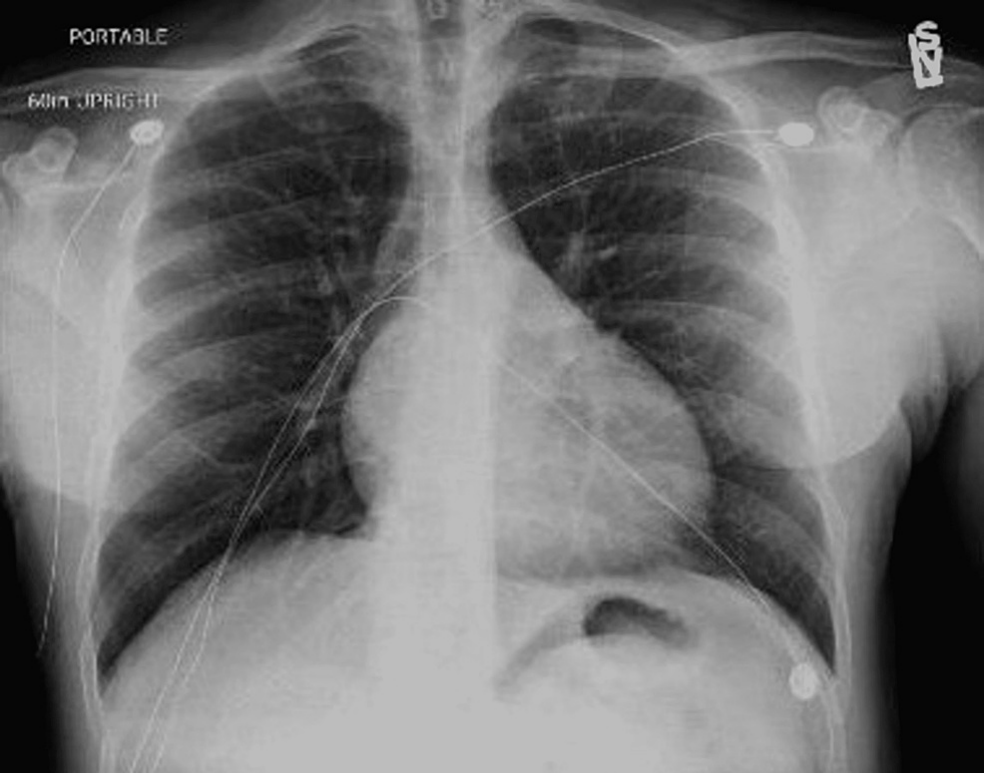
Chest x-ray showing normal cardiac silhouette and lung fields

On presentation, the patient had a blood pressure of 157/105 mmHg and a heart rate of 51/min. He was afebrile at 97.8 F, oxygen saturation was 100% on room air and respiratory rate was 16/min. The initial evaluation was notable for a comfortable middle-aged gentleman, without apparent distress. Cardio-pulmonary examination revealed bilateral equal air entry without any added sounds. There were normal S1 and S2 without any murmurs, pericardial rub, or gallops. There was no chest wall tenderness on palpation. There was no peripheral edema or calf tenderness. An electrocardiogram (ECG) showed sinus bradycardia at 45 bpm without any ST-T changes (Figure 1). A chest X-ray revealed a normal cardiac silhouette and was negative for signs of pulmonary edema (Figure 2). With no previous hospitalization, there was no record of a previous ECG or an image of a chest X-ray for comparison. Initial laboratory investigations revealed a WBC count of 3,100/µL (4,000-11,000/µL), with 37.9 % (25%-33 %) lymphocytes. Hemoglobin was 13.1 g/dL, platelet count was 194,000/µL (150,000-375,000/µL), and renal function tests and electrolytes were within normal limits. Initial troponin-I level was elevated at 0.065 ng/mL (institutional cut-off > 0.032 ng/mL) subsequently trending down on serial testing (0.054 and 0.045 ng/mL) (Table 1). Due to the initial consideration of myocardial ischemia, additional testing for inflammatory markers was not performed.

**Table 1:** Complete blood count, basic metabolic profile, and troponin-I on initial presentation WBC: white blood cell; BUN: blood urea nitrogen; eGFR: estimated glomerular filtration rate

Complete blood count, basic metabolic profile, and troponin-I	
WBC	3,100/µL (4000 -1100/µL)
Neutrophil %	49.4
Lymphocyte %	37.9
Hemoglobin	13.1 g/dl (14-18 g/dl)
Hematocrit	40.2 (40-54)
Platelet	194,000/µL (150,000 – 375,000/µL)
Sodium	144 mmol/L (135-145)
Potassium	3.7 mmol/L (3.6-5.2)
Chloride	102 mmol/L (98-108)
BUN	14 mg/dl (4-22)
Creatinine	1.2 mg/dl (0.6-1.3)
eGFR	84.2 ml/min
Glucose	131 mg/dl (65-99)
Troponin I (institutional cut-off > 0.032 ng/mL)	
0 hours	0.065 ng/mL
3 hours	0.054 ng/mL
8 hours	0.045 ng/mL

**Figure 3:**
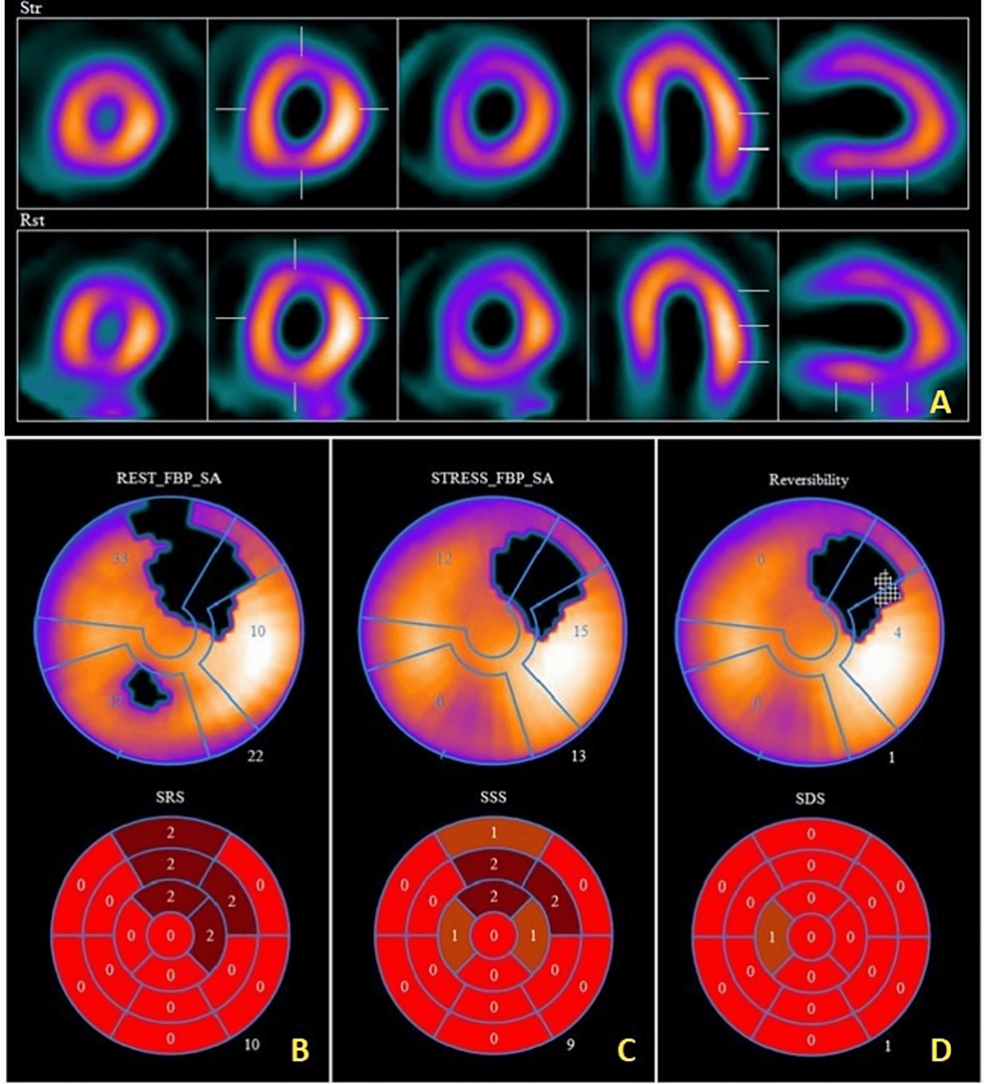
Stress myocardial perfusion image Panel A: Moderate-sized fixed anterior and mid to apical anterolateral defect (sparing the apex) without reversible ischemia; comparative short, vertical, and long-axis images at rest (bottom row) and stress (top row). Panel B, C, D: Comparative polar maps with coronary anatomical (top rows) and 17-segment plots (bottom rows) at rest (panel B), stress (panel C), and reversibility quantification (panel D). SRS: summed rest score; SSS: summed stress score; SDS: summed difference score

With ongoing chest pain and elevated troponin-I, the patient was admitted for an ischemic workup. The transthoracic echocardiogram showed a normal-sized left ventricle with preserved left ventricular ejection fraction of 60-65% without evidence of regional wall motion abnormalities. Right ventricular size and ejection fraction were also preserved and there was no evidence of valvular heart disease or pericardial effusion. Initial differential diagnosis included COVID-19 vaccine-induced myocarditis versus myocardial demand ischemia secondary to previously undiagnosed accelerated hypertension versus ischemic heart disease. Based on the patient’s interval improvement in symptoms after normalization of blood pressure, he was empirically started on dual antiplatelet therapy with aspirin 81 mg daily, and clopidogrel 75 mg daily (to be maintained till planned outpatient stress test), and high-intensity statin therapy with atorvastatin 40 mg at bedtime. Besides initiation of amlodipine, beta-blocker therapy was not administered secondary to the patient’s baseline resting heart rates in the low 60s. With no reported events of myocarditis related to the vaccine at the time and a lack of cardiac MR imaging at the facility, he initially underwent an outpatient treadmill nuclear stress test to rule out an ischemic etiology of his symptoms. He exercised on the treadmill for nine minutes (standard Bruce protocol) achieving a workload of 10.1 METS with normal hemodynamic response to exercise and no ischemic changes or anginal symptoms on the treadmill. Stress myocardial perfusion imaging revealed a moderate-sized fixed anterior and mid to apical anterolateral defect (sparing the apex) without reversible ischemia (Figure 3). 

**Figure 4:**
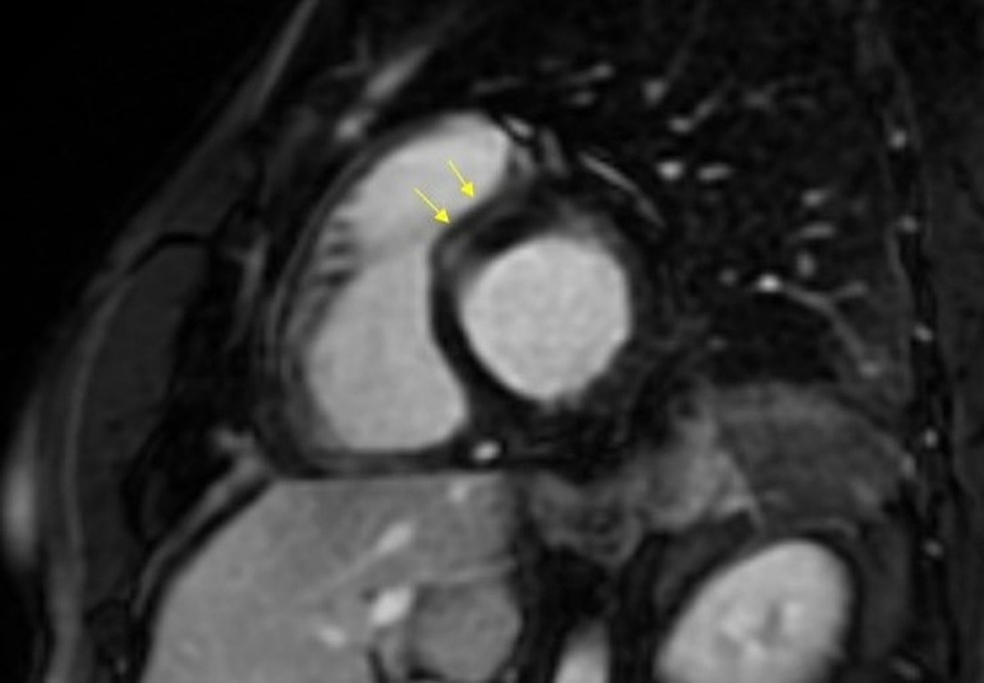
Cardiac MRI with myocardial edema and subtle left ventricular mid-wall late gadolinium enhancement involving the infero septal/inferior wall with preserved left ventricular chamber dimensions and function, consistent with myocarditis.

Due to ambiguity regarding the diagnosis, the defect on stress MPI was deemed to be artifactual in nature, and with recurrent symptoms, the patient was referred for a cardiac MRI (cMRI) with T2-weighted imaging which showed preserved biventricular size and function. There was no evidence of an anterior infarct with study findings notable for the presence of myocardial edema and subtle left ventricular mid-wall late gadolinium enhancement involving the anteroseptal wall at the inferior right ventricular insertion point. With the presence of normal left ventricular chamber size and function, the findings were consistent with myocarditis (Figure 4). Patient's clinical presentation was deemed to be consistent with COVID-19 vaccine-induced myocarditis. Aspirin and clopidogrel were subsequently discontinued. He was initiated on high-dose ibuprofen 600 mg three thrice daily for two weeks followed by three months of colchicine therapy at a dose of 0.6 mg twice daily with interval resolution of his symptoms. Due to the presence of structural heart disease, amlodipine was switched to lisinopril for the management of concomitant hypertension.

## Discussion

Studies have demonstrated an 84% lower risk of reinfection, for seven months following primary COVID-19 infection [7]. However, there is a disparity in the strength and duration of immune responses raised by symptomatic and asymptomatic individuals favoring large-scale vaccination programs [8]. Although patients with COVID-19 infection are at 16 times higher risk of myocarditis compared to patients without COVID-19 infection, the occurrence of clinical myocarditis secondary to COVID-19 infection is rare with subsequent pericardial involvement occurring only in 10% of myocarditis cases [9,10]. The case discussed highlights the occurrence of myocarditis after COVID-19 infection and subsequently after each dose of the COVID-19 vaccine. The patient had no background history suggestive of systemic causes of myocarditis such as infection, toxin, or autoimmune disease. There were, however, no additional symptoms like fever or rash, pointing towards a hypersensitivity reaction. Due to a lack of systemic symptoms, no viral studies were undertaken. The temporal association and periodic recurrence of symptoms consistent with myocarditis after vaccination pointed towards the COVID-19 vaccine as the potential cause.

The pathophysiology of COVID-19 infection that causes cardiovascular manifestations such as acute myocarditis, arrhythmia, ischemic cardiomyopathy, heart failure, pericardial effusion, cardiac tamponade, and thromboembolism has been postulated to occur by a direct or indirect effect on the myocardium and by endothelial dysfunction. Though the causal relationship and underlying mechanism have not been definitively established, the theorized mechanism is this could be secondary to systemic inflammatory response, cytokine storm, or sympathetic activation [3,11]. The SARS-CoV-2 virus has a direct effect on the heart by entry into cardiomyocytes through the angiotensin-converting enzyme 2 receptors which are up-regulated in patients with underlying cardiovascular disease and diabetes mellitus [3]. Viral replication in these infected cardiomyocytes leads to cellular edema and necrosis resulting in contractile dysfunction and myocarditis [12]. In patients with COVID-19 infection and vaccine-related myocarditis, it is difficult to establish if the symptoms are a result of a flare-up after vaccine administration due to incomplete infection resolution.

While a definitive diagnosis of myocarditis can be ascertained by biopsy, troponin elevation secondary to myocardial injury is common in COVID-19 infection and a useful predictor of cardiac involvement and poor outcomes particularly 30-day in-hospital mortality [13,14]. The recommended management for myocarditis is variable and depends on the presence of active COVID-19 infection. The management of COVID-19-associated myocarditis remains conventional. Current guidelines recommend the use of high-dose NSAIDs such as aspirin, ibuprofen, or indomethacin for one to two weeks followed by colchicine for three months in case of acute myocarditis and 6 months of colchicine in case of recurrent myocarditis [15]. In patients who have recovered from COVID-19 infection, cardiac MRI can be utilized for the assessment of cardiac function [16].

The CDC and US-FDA co-managed VAERS, which receive and analyze information on adverse effects of approved vaccines. According to the VAERS, myocarditis is a rare adverse effect of vaccines with 59% of the 708 cases of myocarditis reported by 2018, being secondary to the smallpox vaccine [17]. This adverse effect was noted to be predominant in males aged 19-49 years with onset being less than two weeks post-vaccine administration. The current reporting of COVID-19 vaccine-associated myocarditis represents only the tip of an iceberg. The incidence of cardiac events in the COVID-19 vaccine trials was less than 0.1% [18]. With COVID-19 vaccines being initially approved for emergency authorized use, there is a high likelihood of rarer adverse events going unreported after trials. With the large-scale release and use of vaccines, exposing more cases of myocarditis, the BNT162b2 and mRNA-1273 COVID-19 vaccines are now US-FDA approved, with an added warning related to myocarditis as a potential effect of the mRNA vaccines. 

The life-threatening nature of this condition calls for increased vigilance and a low threshold for evaluation with cMRI in patients vaccinated with the mRNA COVID-19 vaccine. The cMRI diagnostic criteria for myocarditis are well defined, but the limited availability of this diagnostic modality makes early diagnosis challenging resulting in an underrepresentation of vaccine-related myocarditis [4]. In the majority of patients, symptoms of myocarditis are mild and can be detected only by elevated troponin levels [18]. Such patients with mild symptoms can be managed with NSAIDs and must be instructed to abstain from competitive sports for at least three months.

## Conclusions

Myocarditis is an established manifestation of COVID-19 infection and COVID-19 mRNA vaccine. Patients with myocarditis secondary to COVID-19 infection have a possibly higher susceptibility to vaccine-related myocarditis due to an unknown mechanism. The occurrence of myocarditis with COVID-19 infection and recurrence after COVID-19 vaccination could be due to incomplete resolution of primary inflammation. Through this case report, we aim to highlight the potentially higher risk of recurrent myocarditis with COVID-19 vaccine after initial occurrence with COVID-19 infection. We need more studies demonstrating the susceptibility of patients based on age, race, and co-morbidities and this could play a role in the vaccination policy. Studies comparing this adverse effect in patients with previous COVID-19 infection and in uninfected patients are warranted. 
